# Response of Soil Respiration to Grazing in an Alpine Meadow at Three Elevations in Tibet 

**DOI:** 10.1155/2014/265142

**Published:** 2014-03-24

**Authors:** Gang Fu, Xianzhou Zhang, Chengqun Yu, Peili Shi, Yuting Zhou, Yunlong Li, Pengwan Yang, Zhenxi Shen

**Affiliations:** ^1^Chinese Academy of Sciences, Institute of Geographic Sciences and Natural Resources Research, Key Laboratory of Ecosystem Network Observation and Modeling, Lhasa Plateau Ecosystem Research Station, Beijing 100101, China; ^2^Department of Microbiology and Plant Botany, Center for Spatial Analysis, University of Oklahoma, Norman, OK 73019, USA; ^3^College of Resources and Environment, University of Chinese Academy of Sciences, Beijing 100049, China

## Abstract

Alpine meadows are one major type of pastureland on the Tibetan Plateau. However, few studies have evaluated the response of soil respiration (*R*_*s*_) to grazing along an elevation gradient in an alpine meadow on the Tibetan Plateau. Here three fenced enclosures were established in an alpine meadow at three elevations (i.e., 4313 m, 4513 m, and 4693 m) in July 2008. We measured *R*_*s*_ inside and outside the three fenced enclosures in July–September, 2010-2011. Topsoil (0–20 cm) samples were gathered in July, August, and September, 2011. There were no significant differences for *R*_*s*_, dissolved organic C (DOC), and belowground root biomass (BGB) between the grazed and ungrazed soils. Soil respiration was positively correlated with soil organic C (SOC), microbial biomass (MBC), DOC, and BGB. In addition, both *R*_*s*_ and BGB increased with total N (TN), the ratio of SOC to TN, ammonium N (NH_4_^+^-N), and the ratio of NH_4_^+^-N to nitrate N. Our findings suggested that the negligible response of *R*_*s*_ to grazing could be directly attributed to that of respiration substrate and that soil N may indirectly affect *R*_*s*_ by its effect on BGB.

## 1. Introduction 

Soil respiration (*R*_*s*_) is an important flux in the C cycle [1–3]. Raich and Schlesinger [1] indicated that *R*_*s*_ is the second in magnitude to gross primary production but equivalent to or even greater than net primary production. Root and microbial respiration are the two most important components of *R*_*s*_; thus the factors which affect root growth and microbial activity could all influence *R*_*s*_ [4–6]. Water availability and temperature are the two most important abiotic factors controlling *R*_*s*_ at various spatial and temporal scales [7, 8]. The positive relationship between *R*_*s*_ and temperature could be weakened or masked by other factors (e.g., respiration substrate) [9, 10]. Previous studies have shown that *R*_*s*_ increases with respiration substrate, including labile C (e.g., microbial biomass C, MBC; dissolved organic C, DOC) and belowground root biomass (BGB) [5, 10–12]. Soil N also affect *R*_*s*_ by influencing plant growth and microbial activity [6, 13].

Grazing is a major type of land use in grasslands and previous studies have shown inconsistent results on the response of *R*_*s*_ to grazing [14, 15]. Many studies have indicated that grazing significantly decreased *R*_*s*_ [14, 16, 17], whereas other studies have shown quite the contrary result [18, 19]. The responses of soil C and N (e.g., MBC, DOC, microbial biomass N, and dissolved organic N) to grazing differ among previous studies [20–23]. There are also inconsistent results on the response of BGB to grazing [15, 24]. The response of *R*_*s*_ to grazing is complex and may be dependent on the responses of respiration substrate [25] and soil N.

Alpine meadows are a major type of pastureland on the Tibetan Plateau [14, 22] and store 4.68 Pg soil organic C (SOC) with density of 9.05 kg m^−2^ at depth of 0–100 cm [26]. Few studies have evaluated the response of *R*_*s*_ to grazing in an alpine meadow along an elevation gradient, although pasture for domestic sheep and yak is a common land use type on the Tibetan Plateau [22]. Here, we investigated the grazing effect on *R*_*s*_ in an alpine meadow at three elevations (i.e., 4313 m, 4513 m, and 4693 m) on the Northern Tibetan Plateau.

The main objectives of this study were to examine (1) the effect of grazing on *R*_*s*_, soil C, and N and (2) the relationships between *R*_*s*_ and respiration substrate (soil organic C, MBC, DOC, and BGB) and soil N (total N, microbial biomass N, dissolved organic N, ammonium N, and nitrate N) along an elevation gradient in an alpine meadow in Tibet.

## 2. Materials and Methods 

### 2.1. Study Area

The study area (30°30′–30°32′N and 91°03′–91°04′E) was located at the Damxung Grassland Observation Station, Tibetan Autonomous Region in China. Annual mean solar radiation was 7527.6 MJ m^−2^ and sunlight was 2880.9 h [12]. Annual average precipitation was around 476.8 mm and annual potential evapotranspiration was about 1725.7 mm [27]. Annual mean air temperature was 1.3°C [12]. The soil was classified as a shallow sandy loam (~0.5–0.7 m), with organic matter of 0.3–11.2%, total N of 0.03–0.49%, and pH of 6.0–6.7 [22]. The vegetation surrounding the study site was* Kobresia*-dominated alpine meadow [28]. Roots are mainly concentrated in the topsoil layer (0–20 cm) [29].

Based on meteorological observations from 1963 to 2012 at the Damxung Station (4288 m, approximately 4 km from our study site), there was no significant change for annual precipitation, while annual mean air temperature increased at a rate of 0.04°C a^−1^ [12].

### 2.2. Experimental Design

Three sites (about 20 m × 20 m for each) were fenced in an alpine meadow on a south-facing slope on the Nyainqentanglha Mountains along an elevation gradient (i.e., 4313 m, 4513 m, and 4693 m) in July 2008. Before enclosure, the site at elevation 4313 m was winter pasture, while the other two sites were summer pasture [22]. A more detailed description of the experimental design can be found in Fu et al. [22].

Soil temperature (*T*_*s*_) at a depth of 5 cm, soil water content (SWC) at a depth of 10 cm, and air temperature and relative humidity at a height of 15 cm were continuously monitored using data loggers (HOBO weather station, Onset Computer Corporation, USA) at each elevation [22]. Both air temperature and *T*_*s*_ increased with decreasing elevation [22].

### 2.3. Measurement of *R*_*s*_

**Figure 1:**
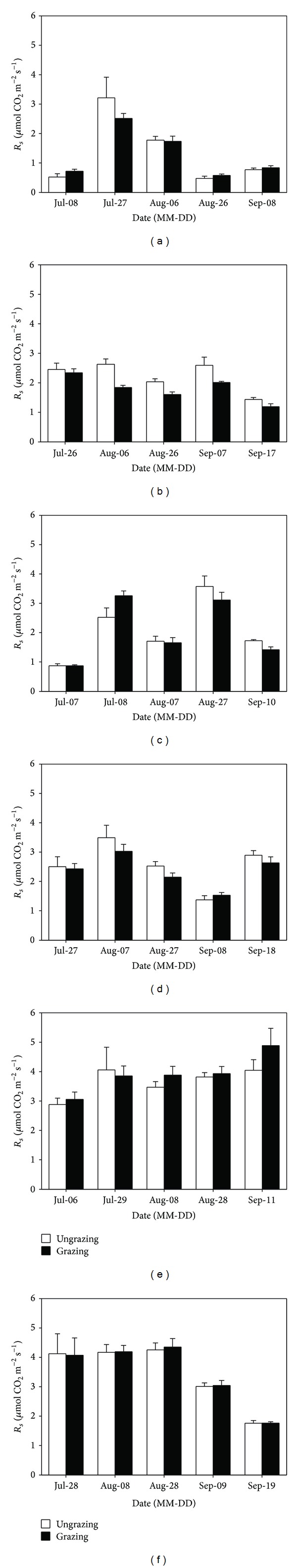
Effects of grazing on soil respiration (*R*_*s*_, *μ*mol CO_2_ m^−2^ s^−1^) in an alpine meadow located at elevations of 4313 m (a, b), 4513 m (c, d), and 4693 m (e, f) on the Tibetan Plateau in 2010 (a, c, and e) and 2011 (b, d, and f), respectively. Error bars represent standard error (*n* = 4).

Soil respiration was measured using a soil CO_2_ flux system (LI-8100, LI-COR Biosciences, Lincoln, NE, USA) [6, 30] during the period from July to September in 2010 and 2011 (Figure 1). Soil respirationat 9:00–11:00 am was close to daily average *R*_*s*_ [6, 31]; thus *R*_*s*_ was measured between 9:00 and 11:00 (local time) in this study. Four polyvinyl chloride (PVC) collars (20 cm in diameter and 5 cm in height) were inserted into the soil to depths of about 2-3 cm on each measuring date. All the PVC collars were installed and the aboveground biomass was removed at least 12 h before *R*_*s*_ measurement in order to reduce disturbance [6, 12]. The opaque survey chamber was manually mounted on PVC collars for *R*_*s*_ measurements [30]. One cycle was performed on each measuring date.

### 2.4. Soil Sampling and Analysis

Topsoil samples (0–20 cm depth) inside and outside the three fenced enclosures were collected (using a soil auger of 3.0 cm in diameter) on July 7, August 9, and September 10, 2011 [22]. Five soil subsamples were randomly sampled and composited into one soil sample for each of the four replicates. The composited soil samples were stored in an icebox and transferred to laboratory. We sieved soil samples (with a sieve of 1 mm diameter) and picked up any visible roots from the sieved soil. Subsamples of the sieved soil were used to measure NO_3_^−^-N, NH_4_^+^-N, DOC, and DON. All the roots in the soil samples were washed, dried at 65°C for 48 h, and weighed.

We extracted 20 g fresh soil samples using 100 mL K_2_SO_4_. The K_2_SO_4_ extracts were filtered through 0.45 *μ*m filter membrane and then soil available N (SAN, NO_3_^−^-N, and NH_4_^+^-N) in the extracts were analyzed on a LACHAT Quickchem Automated Ion Analyzer.

The methods of Jones and Willett [32] were used to determine DOC and dissolved total N (DTN). Briefly, we extracted 20 g fresh soil samples using 100 mL ultrapure water and filtered the extracts through 0.45 *μ*m filter membrane. We analyzed the extractable soil organic C and total N in the ultrapure water extracts using a Liqui TOC II elementar analyzer (Elementar Liqui TOC, Elementar Co., Hanau, Germany) and a UV-1700 PharmaSpec visible spectrophotometer (220 nm and 275 nm), respectively. Dissolved inorganic N (DIN) concentrations in the ultrapure water extracts were also determined on a LACHAT Quickchem Automated Ion Analyzer. Then, DON was calculated as the difference between DTN and DIN. Soil organic C, TN, MBC, and MBN data were obtained from Fu et al. [22].

### 2.5. Statistical Analysis

Repeated-measures analysis of variance (ANOVA) was used to estimate the main and interactive effects of measuring date and grazing on *R*_*s*_ for each site (Table 1). Repeated-measures ANOVA was used to estimate the main and interactive effects of sampling date and grazing on DOC, DON, DOC/DON ratio, NO_3_^−^-N, NH_4_^+^-N, NH_4_^+^-N/NO_3_^−^-N ratio, SAN, and BGB(Table 2). Student-Newman-Keuls multiple comparisons were performed among the three sites. Linear relationships of *R*_*s*_ with SOC, TN, SOC/TN ratio, MBC, MBN, DOC, DON, NH_4_^+^-N, SAN, NH_4_^+^-N/NO_3_^−^-N ratio, and BGB were conducted, respectively. All the statistical tests were performed using the SPSS software (version 16.0; SPSS Inc., Chicago, IL).

## 3. Results 

**Figure 2:**
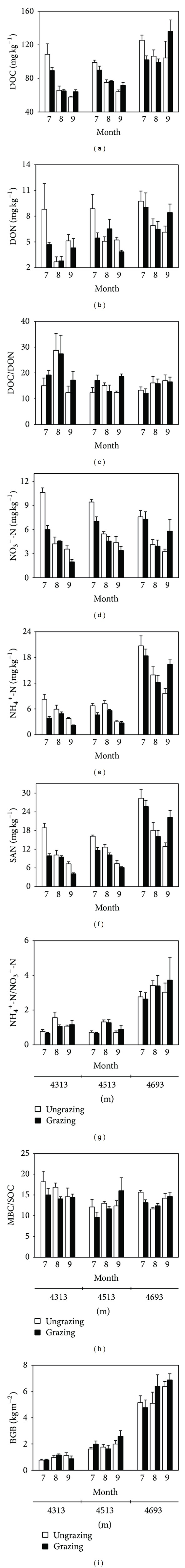
Effects of grazing on dissolved organic C (DOC, mg kg^−1^), dissolved organic N (DON, mg kg^−1^), the ratio of DOC and DON (DOC/DON ratio), nitrate N (NO_3_^−^-N, mg kg^−1^), ammonium N (NH_4_^+^-N, mg kg^−1^), soil available N (SAN, mg kg^−1^), the ratio of NH_4_^+^-N and NO_3_^−^-N (NH_4_^+^-N/NO_3_^−^-N ratio), and belowground root biomass (BGB, kg m^−2^) in an alpine meadow at three elevations (i.e., 4313 m, 4513 m, and 4693 m) on the Tibetan Plateau. Error bars represent standard error (*n* = 4). MBC and SOC data were obtained from Fu et al. [22].

At elevation 4313 m, NO_3_^−^-N, NH_4_^+^-N, and SAN under grazing were 31.78% (1.95 mg kg^−1^), 39.14% (2.34 mg kg^−1^), and 35.41% (4.29 mg kg^−1^) lower compared with ungrazed soils across all the three sampling dates (Figure 2 and Table 2). Similarly, at elevation 4513 m, NO_3_^−^-N, NH_4_^+^-N, and SAN under grazing were 22.00% (1.41 mg kg^−1^), 23.60% (1.33 mg kg^−1^), and 22.75% (2.75 mg kg^−1^) lower than that of ungrazed soils (Figure 2 and Table 2). In contrast, there were no significant differences of NO_3_^−^-N, NH_4_^+^-N, and SAN between ungrazed and grazed soils at elevation 4693 m (Figure 2 and Table 2).

In addition, grazing had no significant effects on NH_4_^+^-N/NO_3_^−^-N ratio, DOC, DON, DOC/DON ratio, and BGB for the three alpine meadow sites (Figure 2 and Table 2). Regardless of grazing, NO_3_^−^-N, NH_4_^+^-N, NH_4_^+^-N/NO_3_^−^-N ratio, SAN, DOC, DON, DOC/DON ratio, and BGB all showed similar seasonal dynamics among the three elevations (Figure 2).

No significant differences of DOC, NH_4_^+^-N, and NH_4_^+^-N/NO_3_^−^-N ratio were found between elevations 4313 m and 4513 m, whereas they were significantly lower compared with elevation 4693 m whether or not grazing was present. Average DOC at elevations 4313 m and 4513 m across all the three sampling dates was 33.01% and 29.31% lower than that of elevation 4693 m, respectively, irrespective of grazing (*P* < 0.05). Average NH_4_^+^-N at elevations 4313 m and 4513 m was 68.40% and 67.23% lower compared with elevation 4693 m, respectively (*P* < 0.05). Average NH_4_^+^-N/NO_3_^−^-N ratio at elevations 4313 m and 4513 m was 66.76% and 70.52% lower compared with elevation 4693 m, respectively (*P* < 0.05).

Average DON at elevations 4313 m and 4513 m across all the three sampling dates was 50.78% and 33.84% lower than that of elevation 4693 m under grazing, respectively (*P* < 0.05), whereas there was no significant difference between elevations 4313 m and 4513 m. There were no significant differences of average DON among the three sites when grazing was absent.

Average SAN at elevations 4313 m and 4513 m across all the three sampling dates was 38.69% and 38.88% lower than that of elevation 4693 m when grazing was absent, respectively (*P* < 0.05), while no significant difference between the two lower elevations was found. By contrast, average SAN increased with increasing elevation under grazing (*F* = 375.30, *P* < 0.001).

No significant differences of DOC/DON ratio and NO_3_^−^-N were found among the three sites whether or not grazing was present.

The main effect of grazing and its interactive effect with measuring date on *R*_*s*_ were not significant for each alpine meadow site (Figure 1 and Table 1). Grazing only tended to decrease the average *R*_*s*_ across all the measuring dates by 14.02% (0.25 *μ*mol CO_2_ m^−2^ s^−1^), 4.70% (0.11 *μ*mol CO_2_ m^−2^ s^−1^), and −4.07% (−0.15 *μ*mol CO_2_ m^−2^ s^−1^) at elevations 4313 m, 4513 m, and 4693 m, respectively. In contrast, there was significant seasonal variation for *R*_*s*_ (Figure 1 and Table 1). Regardless of grazing, *R*_*s*_ showed similar seasonal dynamics among the three elevations (Figure 1).

There were significant elevation effects on *R*_*s*_ (*F* = 147.94, *P* < 0.001 for ungrazed condition; *F* = 227.25, *P* < 0.001 for grazed condition) and BGB (*F* = 315.20, *P* < 0.001 for ungrazed condition; *F* = 58.81, *P* < 0.001 for grazed condition) across all the measuring dates.

**Figure 3:**
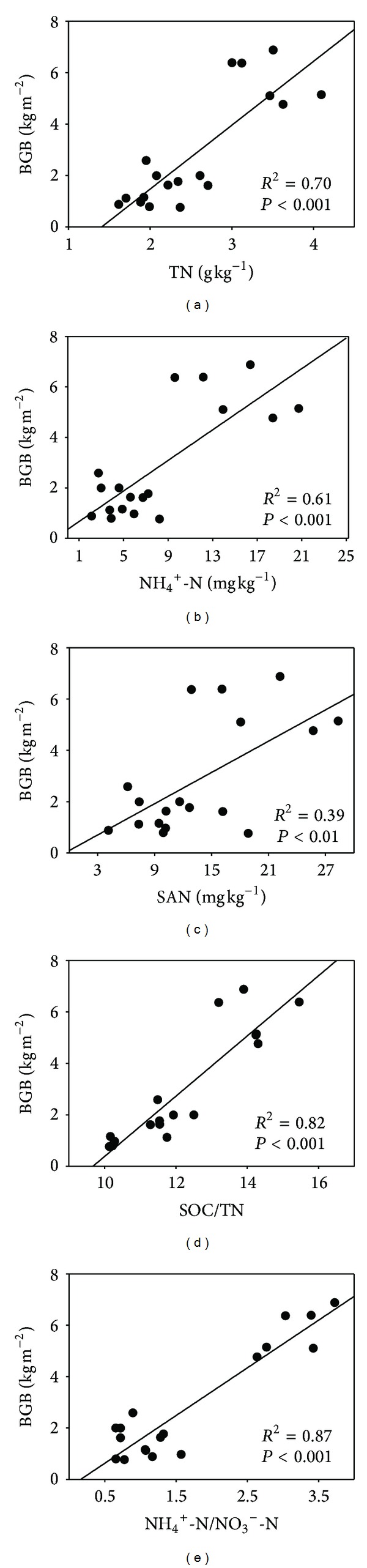
Relationships between belowground root biomass (BGB, kg m^−2^) and total N (TN, g kg^−1^), ammonium N (NH_4_^+^-N, mg kg^−1^), soil available N (SAN, mg kg^−1^), the ratio of SOC and TN (SOC/TN ratio), and the ratio of NH_4_^+^-N and nitrate N (NH_4_^+^-N/NO_3_^−^-N ratio), respectively. SOC and TN data were obtained from Fu et al. [22].

Belowground biomass was positively related to TN, NH_4_^+^-N, SAN, SOC/TN ratio, and NH_4_^+^-N/NO_3_^−^-N ratio, respectively (Figure 3), but not to NO_3_^−^-N (data not shown).

**Figure 4:**
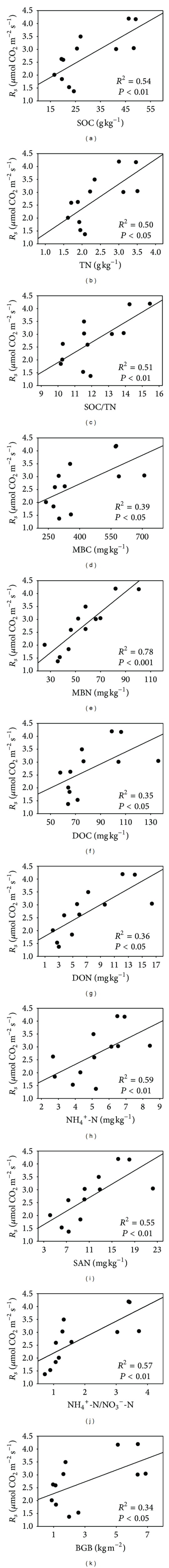
Relationships between soil respiration (*R*_*s*_, µmol CO_2_ m^−2^ s^−1^) measured early in August and September, 2011, and soil organic C (SOC, g kg^−1^), total N (TN, g kg^−1^), the ratio of SOC and TN (SOC/TN ratio), microbial biomass C (MBC, mg kg^−1^), microbial biomass N (MBN, mg kg^−1^), dissolved organic C (DOC, mg kg^−1^), dissolved organic N (DON, mg kg^−1^), ammonium N (NH_4_^+^-N, mg kg^−1^), soil available N (SAN, mg kg^−1^), the ratio of NH_4_^+^-N and nitrate N (NH_4_^+^-N/NO_3_^−^-N ratio), and belowground root biomass (BGB, kg m^−2^), respectively. SOC, TN, MBC, and MBN data were obtained from Fu et al. [22].

*R*_*s*_ was positively correlated with SOC, TN, SOC/TN ratio, MBC, MBN, DOC, DON, NH_4_^+^-N, SAN, NH_4_^+^-N/NO_3_^−^-N ratio, and BGB, respectively (Figure 4). However, *R*_*s*_ was not linearly correlated with DOC/DON ratio, MBC/MBN ratio, and NO_3_^−^-N (data not shown).

## 4. Discussion 

Previous studies indicated that grazer urine and dung stimulated soil microbial activity and accelerated nutrient cycling in grasslands [33, 34]. However, this effect may be often weakened at the three alpine meadow sites because the dung of yak and goat was removed by local residents.

Generally, grazing did not alter the distributions of *R*_*s*_, DOC, DON, DOC/DON ratio, NO_3_^−^-N, NH_4_^+^-N, and BGB along the elevation gradient, which was in line with previous studies [22, 35, 36].

Soil microbial biomass N at elevations 4313 m and 4693 m and SAN (including NO_3_^−^-N and NH_4_^+^-N) at elevations 4313 m and 4513 m under grazing were significantly lower compared with ungrazed soils, while there were no significant differences of SOC, TN, DOC, DON, BGB, and *R*_*s*_ between grazed and ungrazed soils (Tables 1 and 2, [22]). This suggests that soil microbial biomass and available N may respond more rapidly to grazing than SOC, TN, DOC, DON, BGB, and *R*_*s*_. The negligible response of *R*_*s*_ to grazing was consistent with some previous studies conducted on the Tibetan Plateau (e.g., [15]).

Previous studies showed that BGB increased with increasing TN in alpine grasslands on the Tibetan Plateau [36, 37]. Our study confirmed this finding (Figure 3). Besides, BGB increased with increasing NH_4_^+^-N, SAN, and NH_4_^+^-N/NO_3_^−^-N ratio, but not with NO_3_^−^-N. Therefore, the positive relationship between BGB and SAN may be mainly attributed to that between BGB and NH_4_^+^-N. In addition, the ratio of different soil available N forms could affect BGB.

Similar to BGB, *R*_*s*_ increased with TN, SOC/TN ratio, NH_4_^+^-N, SAN, and NH_4_^+^-N/NO_3_^−^-N ratio, respectively (Figure 4). Meanwhile, *R*_*s*_ was positively correlated with BGB (Figure 4). Therefore, the effect of soil N availability and form on *R*_*s*_ was probably associated with the effect of soil N availability and form on BGB. In addition, the negligible response of *R*_*s*_ to grazing may be directly attributed to that of SOC, MBC, DOC, and BGB.

The positive relationships between DOC and SOC, DON and TN, DOC and MBC, DON and MBN, and SAN and MBN (data not shown) were in accordance with previous studies which were made in alpine meadows on the Tibetan Plateau [22, 38] and an upland grassland of northern England [39]. Previous studies found that DOC was a good index in reflecting C availability of soil microorganisms [40, 41]. Therefore, the variation of soil microbial biomass along the elevation gradient may be not only associated with that of SOC and TN [22] but also with that of DOC and DON [6, 11, 42]. In other words, soil microbial activity may regulate the balances of soil inorganic and organic C and N pools in this alpine meadow.

Many studies have found the positive relationship between *R*_*s*_ and *T*_*s*_ in various ecosystems [3, 15]. In contrast, *R*_*s*_ increased significantly with increasing elevation, while both soil and air temperatures declined in the current study. In other words, the relationship between *R*_*s*_ and *T*_*s*_ was negative along the elevation gradient. This implied that other factors (e.g., respiration substrate) probably regulated or confounded the positive relationship between respiration and temperature [10, 25]. This viewpoint was as confirmed by the positive relationships between *R*_*s*_ and BGB, SOC, MBC, and DOC (Figure 4).

## 5. Conclusions 

In this study, we measured soil respiration under grazed and ungrazed conditions in an alpine meadow along an elevation gradient (4313–4693 m with approximate 200 m interval) in Tibet during the period from July to September in 2010-2011. We found that grazing did not significantly affect soil respiration, which was probably attributed to the insignificant response of respiration substrate (e.g., soil organic C and belowground root biomass) to grazing. Soil N availability and the ratio of ammonium to nitrate N might also influence soil respiration by affecting belowground root growth.

## Figures and Tables

**Table 1:** Repeated-measures analysis of variance for the main and interactive effects of grazing (G) and measuring date (D) on soil respiration (*R*_*s*_, µmol CO_2_ m^−2^ s^−1^) in an alpine meadow at three elevations (i.e., 4313 m, 4513 m, and 4693 m) on the Tibetan Plateau (*n* = 4).

Model	4313 m		4513 m		4693 m	
	*F *	*P *	*F *	*P *	*F *	*P *
G	5.94	0.051	2.39	0.173	1.22	0.311
D	**34.12 **	<0.001	**29.08 **	<0.001	**10.02 **	<0.001
G × D	1.60	0.249	1.39	0.277	0.31	0.837

**Table 2:** Repeated-measures analysis of variance for the main and interactive effects of grazing (G) and sampling date (D) on dissolved organic C (DOC, mg kg^−1^) and N (DON, mg kg^−1^), the ratio of DOC and DON (DOC/DON ratio), nitrate N (NO_3_^−^-N, mg kg^−1^), ammonium N (NH_4_^+^-N, mg kg^−1^), the ratio of NH_4_^+^-N and NO_3_^−^-N (NH_4_^+^-N/NO_3_^−^-N ratio), soil available N (SAN, mg kg^−1^), and belowground root biomass (BGB, kg m^−2^) in an alpine meadow at three elevations (i.e., 4313 m, 4513 m, and 4693 m) on the Tibetan Plateau (*n* = 4).

Elevation	Model	DOC	DON	DOC/DON ratio	NO_3_^−^-N	NH_4_^+^-N	NH_4_^+^-N/NO_3_^−^-N ratio	SAN	BGB
4313 m	G	0.77	1.65	0.46	27.10**	26.05**	1.05	95.61***	0.00
	D	26.25***	4.80*	5.00*	57.55***	10.47**	8.95**	29.95***	2.79
	G × D	2.64	1.46	0.29	11.02**	3.00	2.16	7.13*	1.45

4513 m	G	0.00	2.23	5.51	7.29*	13.99**	0.04	16.50**	1.52
	D	35.53***	4.38*	0.40	51.70***	29.87***	12.29***	57.31***	2.74
	G × D	3.15	3.81	3.49	1.85	2.05	0.51	3.07	0.98

4693 m	G	0.00	0.16	0.12	0.82	0.27	0.08	0.65	0.64
	D	1.33	3.67	3.03	9.24**	15.27***	0.94	15.78***	3.85
	G × D	3.41	1.24	0.04	1.79	6.95**	0.30	5.67*	0.95

*, **, and ***means *P* < 0.05, *P* < 0.01, and *P* < 0.001, respectively.
